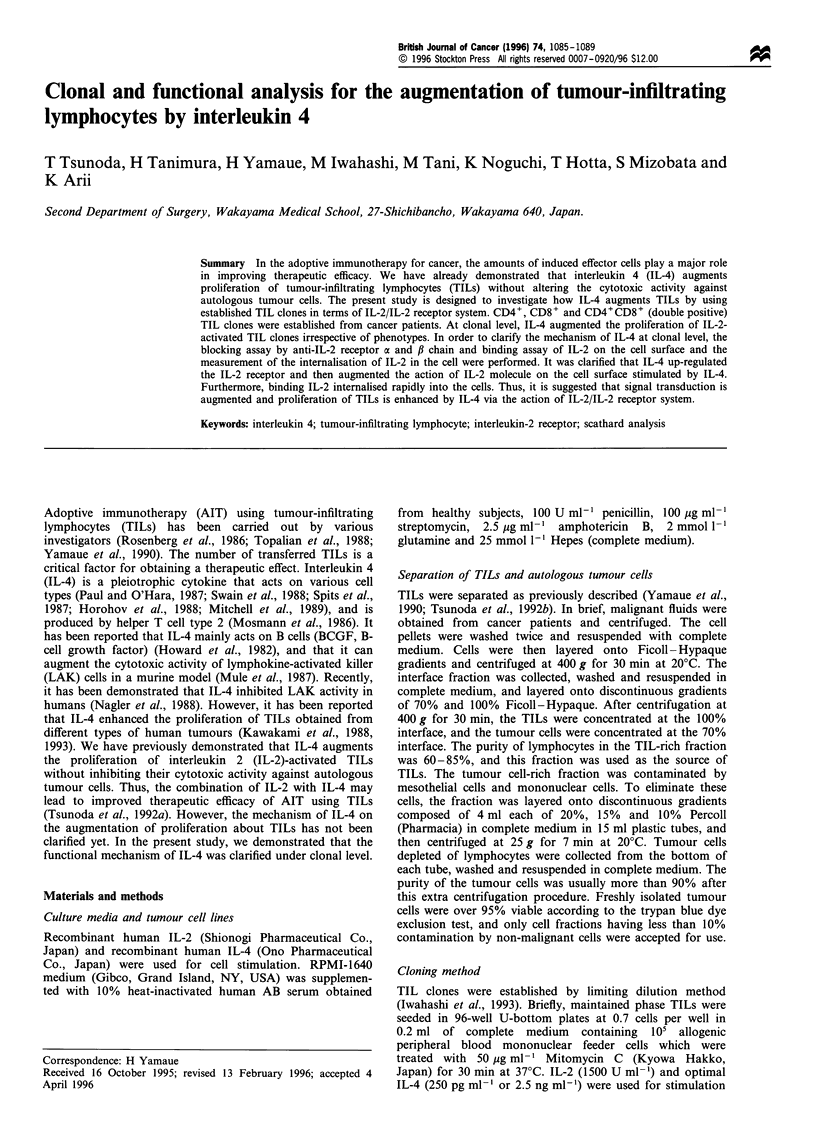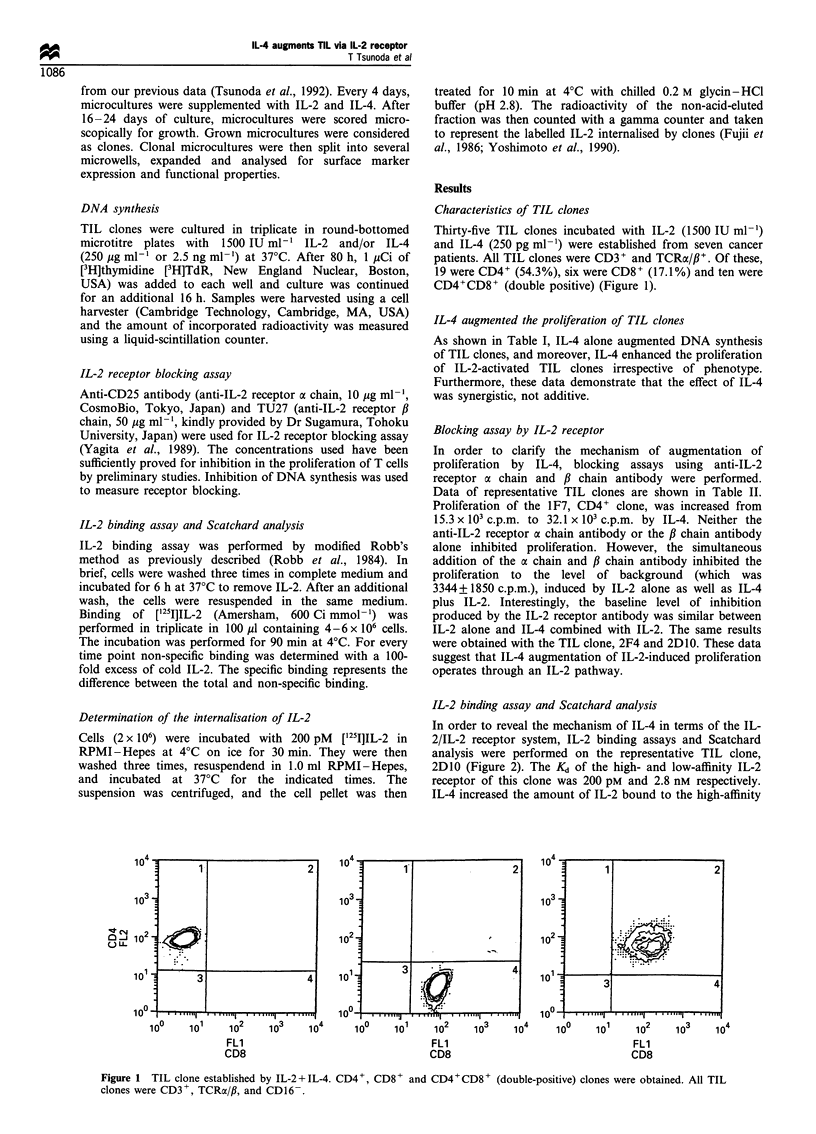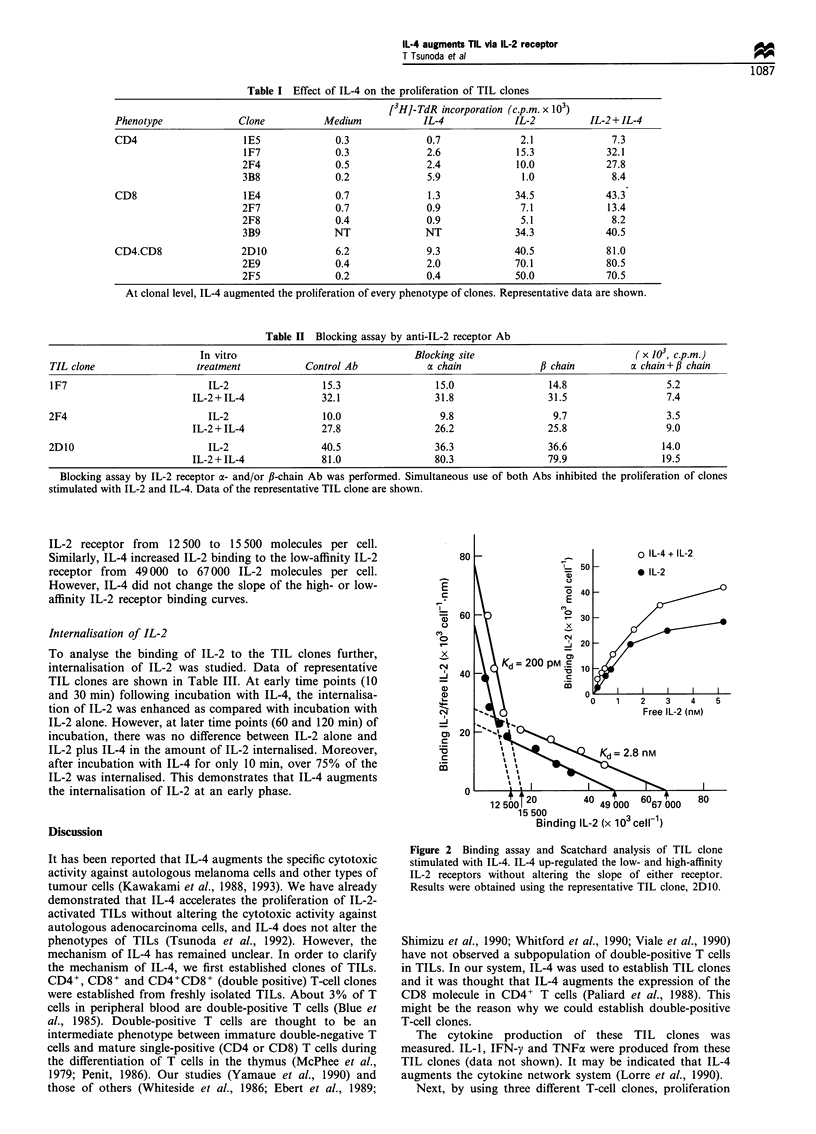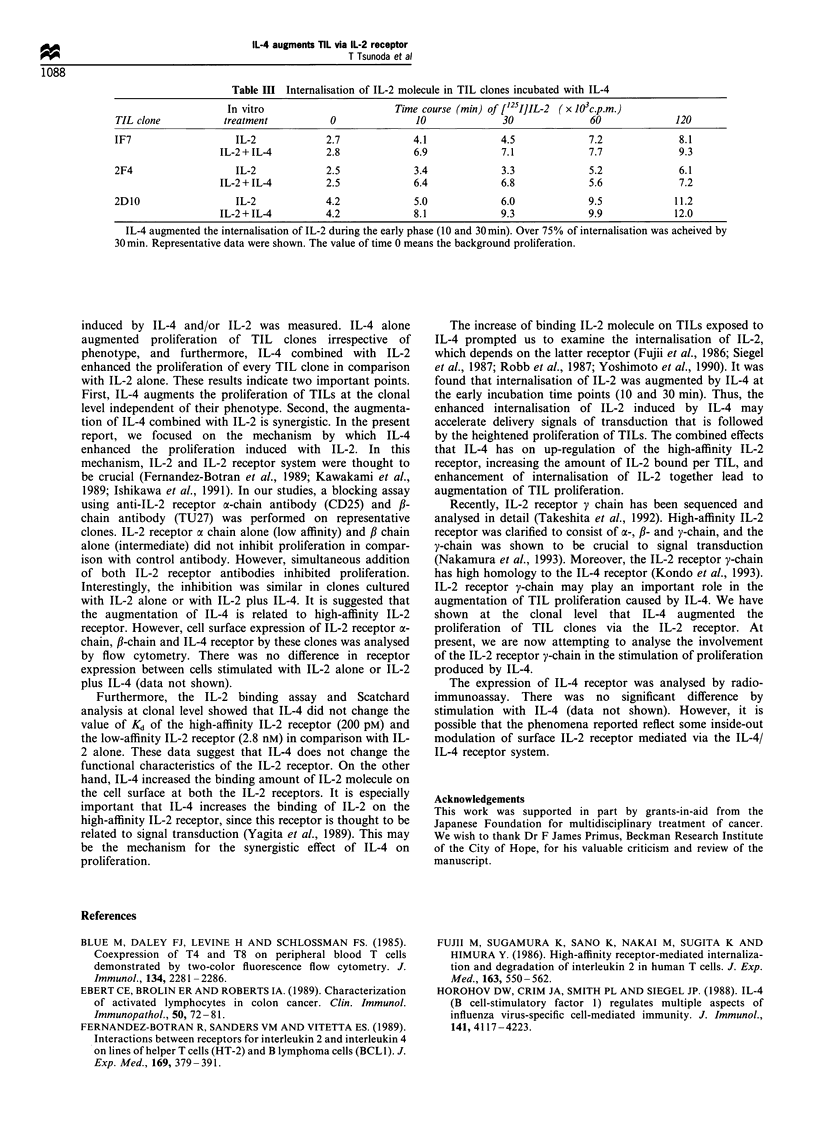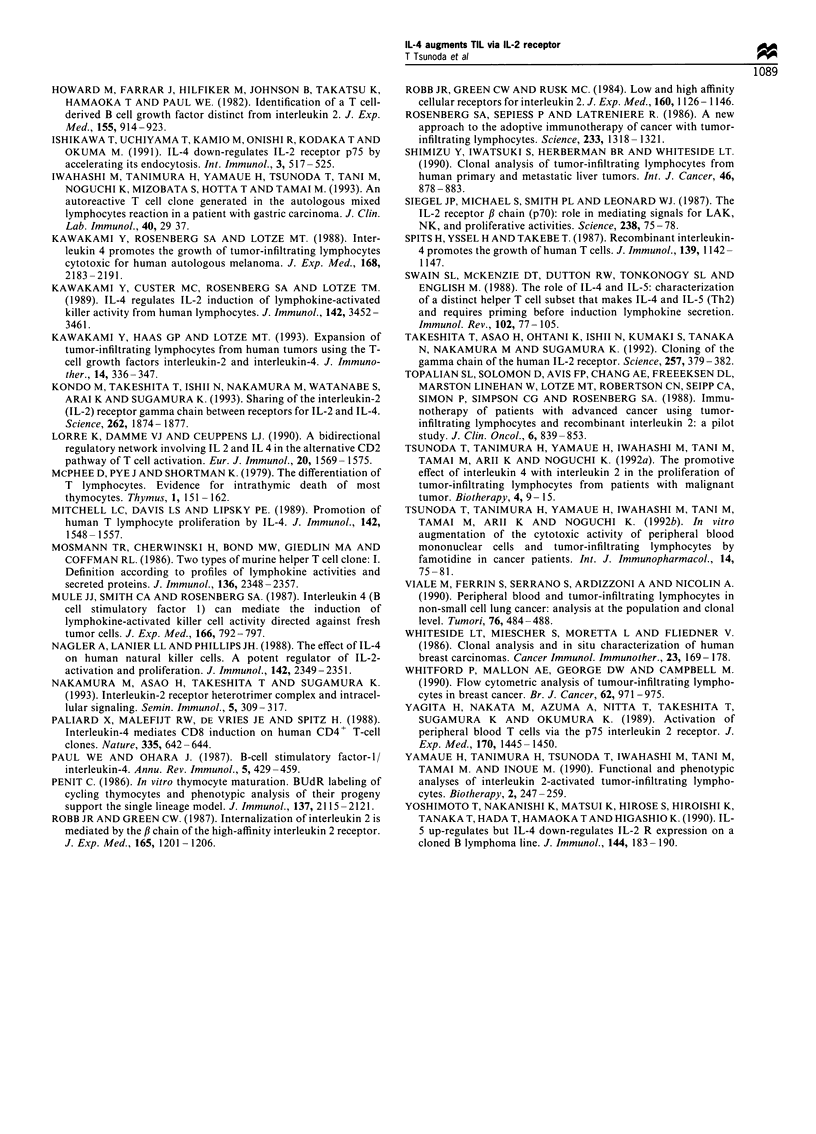# Clonal and functional analysis for the augmentation of tumour-infiltrating lymphocytes by interleukin 4.

**DOI:** 10.1038/bjc.1996.494

**Published:** 1996-10

**Authors:** T. Tsunoda, H. Tanimura, H. Yamaue, M. Iwahashi, M. Tani, K. Noguchi, T. Hotta, S. Mizobata, K. Arii

**Affiliations:** Second Department of Surgery, Wakayama Medical School, Japan.

## Abstract

In the adoptive immunotherapy for cancer, the amounts of induced effector cells play a major role in improving therapeutic efficacy. We have already demonstrated that interleukin 4 (IL-4) augments proliferation of tumour-infiltrating lymphocytes (TILs) without altering the cytotoxic activity against autologous tumour cells. The present study is designed to investigate how IL-4 augments TILs by using established TIL clones in terms of IL-2/IL-2 receptor system. CD4+, CD8+ and CD4+ CD8+ (double positive) TIL clones were established from cancer patients. At clonal level, IL-4 augmented the proliferation of IL-2-activated TIL clones irrespective of phenotypes. In order to clarify the mechanism of IL-4 at clonal level, the blocking assay by anti-IL-2 receptor alpha and beta chain and binding assay of IL-2 on the cell surface and the measurement of the internalisation of IL-2 in the cell were performed. It was clarified that IL-4 up-regulated the IL-2 receptor and then augmented the action of IL-2 molecule on the cell surface stimulated by IL-4. Furthermore, binding IL-2 internalised rapidly into the cells. Thus, it is suggested that signal transduction is augmented and proliferation of TILs is enhanced by IL-4 via the action of IL-2/IL-2 receptor system.


British Journal of Cancer (1996) 74, 1085-1089
© 1996 Stockton Press All rights reserved 0007-0920/96 $12.00

### Clonal and functional analysis for the augmentation of tumour-infiltrating lymphocytes by interleukin 4

T Tsunoda, H Tanimura, H Yamaue, M Iwahashi, M Tani, K Noguchi, T Hotta, S Mizobata and K Arii

*Second Department of Surgery, Wakayama Medical School, 27-Shichibancho, Wakayama 640, Japan.*

**Summary** In the adoptive immunotherapy for cancer, the amounts of induced effector cells play a major role in improving therapeutic efficacy. We have already demonstrated that interleukin 4 (IL-4) augments proliferation of tumour-infiltrating lymphocytes (TILs) without altering the cytotoxic activity against autologous tumour cells. The present study is designed to investigate how IL-4 augments TILs by using established TIL clones in terms of IL-2/IL-2 receptor system. $CD4^+$, $CD8^+$ and $CD4^+CD8^+$ (double positive) TIL clones were established from cancer patients. At clonal level, IL-4 augmented the proliferation of IL-2-activated TIL clones irrespective of phenotypes. In order to clarify the mechanism of IL-4 at clonal level, the blocking assay by anti-IL-2 receptor $\alpha$ and $\beta$ chain and binding assay of IL-2 on the cell surface and the measurement of the internalisation of IL-2 in the cell were performed. It was clarified that IL-4 up-regulated the IL-2 receptor and then augmented the action of IL-2 molecule on the cell surface stimulated by IL-4. Furthermore, binding IL-2 internalised rapidly into the cells. Thus, it is suggested that signal transduction is augmented and proliferation of TILs is enhanced by IL-4 via the action of IL-2/IL-2 receptor system.

**Keywords:** interleukin 4; tumour-infiltrating lymphocyte; interleukin-2 receptor; scathard analysis

Adoptive immunotherapy (AIT) using tumour-infiltrating lymphocytes (TILs) has been carried out by various investigators (Rosenberg *et al.*, 1986; Topalian *et al.*, 1988; Yamaue *et al.*, 1990). The number of transferred TILs is a critical factor for obtaining a therapeutic effect. Interleukin 4 (IL-4) is a pleiotrophic cytokine that acts on various cell types (Paul and O'Hara, 1987; Swain *et al.*, 1988; Spits *et al.*, 1987; Horohov *et al.*, 1988; Mitchell *et al.*, 1989), and is produced by helper T cell type 2 (Mosmann *et al.*, 1986). It has been reported that IL-4 mainly acts on B cells (BCGF, B-cell growth factor) (Howard *et al.*, 1982), and that it can augment the cytotoxic activity of lymphokine-activated killer (LAK) cells in a murine model (Mule *et al.*, 1987). Recently, it has been demonstrated that IL-4 inhibited LAK activity in humans (Nagler *et al.*, 1988). However, it has been reported that IL-4 enhanced the proliferation of TILs obtained from different types of human tumours (Kawakami *et al.*, 1988, 1993). We have previously demonstrated that IL-4 augments the proliferation of interleukin 2 (IL-2)-activated TILs without inhibiting their cytotoxic activity against autologous tumour cells. Thus, the combination of IL-2 with IL-4 may lead to improved therapeutic efficacy of AIT using TILs (Tsunoda *et al.*, 1992*a*). However, the mechanism of IL-4 on the augmentation of proliferation about TILs has not been clarified yet. In the present study, we demonstrated that the functional mechanism of IL-4 was clarified under clonal level.

### Materials and methods

### *Culture media and tumour cell lines*

Recombinant human IL-2 (Shionogi Pharmaceutical Co., Japan) and recombinant human IL-4 (Ono Pharmaceutical Co., Japan) were used for cell stimulation. RPMI-1640 medium (Gibco, Grand Island, NY, USA) was supplemented with 10% heat-inactivated human AB serum obtained from healthy subjects, 100 U $ml^{-1}$ penicillin, 100 $\mu g$ $ml^{-1}$ streptomycin, 2.5 $\mu g$ $ml^{-1}$ amphotericin B, 2 mmol $l^{-1}$ glutamine and 25 mmol $l^{-1}$ Hepes (complete medium).

### *Separation of TILs and autologous tumour cells*

TILs were separated as previously described (Yamaue *et al.*, 1990; Tsunoda *et al.*, 1992*b*). In brief, malignant fluids were obtained from cancer patients and centrifuged. The cell pellets were washed twice and resuspended with complete medium. Cells were then layered onto Ficoll–Hypaque gradients and centrifuged at 400 *g* for 30 min at 20°C. The interface fraction was collected, washed and resuspended in complete medium, and layered onto discontinuous gradients of 70% and 100% Ficoll–Hypaque. After centrifugation at 400 *g* for 30 min, the TILs were concentrated at the 100% interface, and the tumour cells were concentrated at the 70% interface. The purity of lymphocytes in the TIL-rich fraction was 60–85%, and this fraction was used as the source of TILs. The tumour cell-rich fraction was contaminated by mesothelial cells and mononuclear cells. To eliminate these cells, the fraction was layered onto discontinuous gradients composed of 4 ml each of 20%, 15% and 10% Percoll (Pharmacia) in complete medium in 15 ml plastic tubes, and then centrifuged at 25 *g* for 7 min at 20°C. Tumour cells depleted of lymphocytes were collected from the bottom of each tube, washed and resuspended in complete medium. The purity of the tumour cells was usually more than 90% after this extra centrifugation procedure. Freshly isolated tumour cells were over 95% viable according to the trypan blue dye exclusion test, and only cell fractions having less than 10% contamination by non-malignant cells were accepted for use.

### *Cloning method*

TIL clones were established by limiting dilution method (Iwahashi *et al.*, 1993). Briefly, maintained phase TILs were seeded in 96-well U-bottom plates at 0.7 cells per well in 0.2 ml of complete medium containing $10^5$ allogenic peripheral blood mononuclear feeder cells which were treated with 50 $\mu g$ $ml^{-1}$ Mitomycin C (Kyowa Hakko, Japan) for 30 min at 37°C. IL-2 (1500 U $ml^{-1}$) and optimal IL-4 (250 pg $ml^{-1}$ or 2.5 ng $ml^{-1}$) were used for stimulation

Correspondence: H Yamaue
Received 16 October 1995; revised 13 February 1996; accepted 4 April 1996

from our previous data (Tsunoda et al., 1992). Every 4 days, microcultures were supplemented with IL-2 and IL-4. After 16–24 days of culture, microcultures were scored microscopically for growth. Grown microcultures were considered as clones. Clonal microcultures were then split into several microwells, expanded and analysed for surface marker expression and functional properties.

*DNA synthesis*

TIL clones were cultured in triplicate in round-bottomed microtitre plates with 1500 IU $ml^{-1}$ IL-2 and/or IL-4 (250 $\mu g$ $ml^{-1}$ or 2.5 ng $ml^{-1}$) at 37°C. After 80 h, 1 $\mu Ci$ of [$^{3}H$]thymidine [$^{3}H$]TdR, New England Nuclear, Boston, USA) was added to each well and culture was continued for an additional 16 h. Samples were harvested using a cell harvester (Cambridge Technology, Cambridge, MA, USA) and the amount of incorporated radioactivity was measured using a liquid-scintillation counter.

*IL-2 receptor blocking assay*

Anti-CD25 antibody (anti-IL-2 receptor $\alpha$ chain, 10 $\mu g$ $ml^{-1}$, CosmoBio, Tokyo, Japan) and TU27 (anti-IL-2 receptor $\beta$ chain, 50 $\mu g$ $ml^{-1}$, kindly provided by Dr Sugamura, Tohoku University, Japan) were used for IL-2 receptor blocking assay (Yagita et al., 1989). The concentrations used have been sufficiently proved for inhibition in the proliferation of T cells by preliminary studies. Inhibition of DNA synthesis was used to measure receptor blocking.

*IL-2 binding assay and Scatchard analysis*

IL-2 binding assay was performed by modified Robb's method as previously described (Robb et al., 1984). In brief, cells were washed three times in complete medium and incubated for 6 h at 37°C to remove IL-2. After an additional wash, the cells were resuspended in the same medium. Binding of [$^{125}I$]IL-2 (Amersham, 600 Ci $mmol^{-1}$) was performed in triplicate in 100 $\mu l$ containing $4-6 \times 10^{6}$ cells. The incubation was performed for 90 min at 4°C. For every time point non-specific binding was determined with a 100-fold excess of cold IL-2. The specific binding represents the difference between the total and non-specific binding.

*Determination of the internalisation of IL-2*

Cells ($2 \times 10^{6}$) were incubated with 200 pM [$^{125}I$]IL-2 in RPMI–Hepes at 4°C on ice for 30 min. They were then washed three times, resuspendend in 1.0 ml RPMI–Hepes, and incubated at 37°C for the indicated times. The suspension was centrifuged, and the cell pellet was then treated for 10 min at 4°C with chilled 0.2 M glycin–HCl buffer (pH 2.8). The radioactivity of the non-acid-eluted fraction was then counted with a gamma counter and taken to represent the labelled IL-2 internalised by clones (Fujii et al., 1986; Yoshimoto et al., 1990).

### Results

*Characteristics of TIL clones*

Thirty-five TIL clones incubated with IL-2 (1500 IU $ml^{-1}$) and IL-4 (250 pg $ml^{-1}$) were established from seven cancer patients. All TIL clones were $CD3^{+}$ and $TCR\alpha/\beta^{+}$. Of these, 19 were $CD4^{+}$ (54.3%), six were $CD8^{+}$ (17.1%) and ten were $CD4^{+}CD8^{+}$ (double positive) (Figure 1).

*IL-4 augmented the proliferation of TIL clones*

As shown in Table I, IL-4 alone augmented DNA synthesis of TIL clones, and moreover, IL-4 enhanced the proliferation of IL-2-activated TIL clones irrespective of phenotype. Furthermore, these data demonstrate that the effect of IL-4 was synergistic, not additive.

*Blocking assay by IL-2 receptor*

In order to clarify the mechanism of augmentation of proliferation by IL-4, blocking assays using anti-IL-2 receptor $\alpha$ chain and $\beta$ chain antibody were performed. Data of representative TIL clones are shown in Table II. Proliferation of the 1F7, $CD4^{+}$ clone, was increased from $15.3 \times 10^{3}$ c.p.m. to $32.1 \times 10^{3}$ c.p.m. by IL-4. Neither the anti-IL-2 receptor $\alpha$ chain antibody or the $\beta$ chain antibody alone inhibited proliferation. However, the simultaneous addition of the $\alpha$ chain and $\beta$ chain antibody inhibited the proliferation to the level of background (which was $3344 \pm 1850$ c.p.m.), induced by IL-2 alone as well as IL-4 plus IL-2. Interestingly, the baseline level of inhibition produced by the IL-2 receptor antibody was similar between IL-2 alone and IL-4 combined with IL-2. The same results were obtained with the TIL clone, 2F4 and 2D10. These data suggest that IL-4 augmentation of IL-2-induced proliferation operates through an IL-2 pathway.

*IL-2 binding assay and Scatchard analysis*

In order to reveal the mechanism of IL-4 in terms of the IL-2/IL-2 receptor system, IL-2 binding assays and Scatchard analysis were performed on the representative TIL clone, 2D10 (Figure 2). The $K_d$ of the high- and low-affinity IL-2 receptor of this clone was 200 pM and 2.8 nM respectively. IL-4 increased the amount of IL-2 bound to the high-affinity

**Figure 1** TIL clone established by IL-2+IL-4. $CD4^{+}$, $CD8^{+}$ and $CD4^{+}CD8^{+}$ (double-positive) clones were obtained. All TIL clones were $CD3^{+}$, $TCR\alpha/\beta$, and $CD16^{-}$.

**Table I** Effect of IL-4 on the proliferation of TIL clones

| *Phenotype* | *Clone* | *Medium* | *[$^3H$]-TdR incorporation (c.p.m. × $10^3$)* *IL-4* | *IL-2* | *IL-2+IL-4* |
|---|---|---|---|---|---|
| CD4 | 1E5 | 0.3 | 0.7 | 2.1 | 7.3 |
| | 1F7 | 0.3 | 2.6 | 15.3 | 32.1 |
| | 2F4 | 0.5 | 2.4 | 10.0 | 27.8 |
| | 3B8 | 0.2 | 5.9 | 1.0 | 8.4 |
| CD8 | 1E4 | 0.7 | 1.3 | 34.5 | 43.3 |
| | 2F7 | 0.7 | 0.9 | 7.1 | 13.4 |
| | 2F8 | 0.4 | 0.9 | 5.1 | 8.2 |
| | 3B9 | NT | NT | 34.3 | 40.5 |
| CD4.CD8 | 2D10 | 6.2 | 9.3 | 40.5 | 81.0 |
| | 2E9 | 0.4 | 2.0 | 70.1 | 80.5 |
| | 2F5 | 0.2 | 0.4 | 50.0 | 70.5 |

At clonal level, IL-4 augmented the proliferation of every phenotype of clones. Representative data are shown.

**Table II** Blocking assay by anti-IL-2 receptor Ab

| *TIL clone* | In vitro *treatment* | *Control Ab* | *Blocking site* *α chain* | *β chain* | *( × $10^3$, c.p.m.)* *α chain + β chain* |
|---|---|---|---|---|---|
| 1F7 | IL-2 | 15.3 | 15.0 | 14.8 | 5.2 |
| | IL-2+IL-4 | 32.1 | 31.8 | 31.5 | 7.4 |
| 2F4 | IL-2 | 10.0 | 9.8 | 9.7 | 3.5 |
| | IL-2+IL-4 | 27.8 | 26.2 | 25.8 | 9.0 |
| 2D10 | IL-2 | 40.5 | 36.3 | 36.6 | 14.0 |
| | IL-2+IL-4 | 81.0 | 80.3 | 79.9 | 19.5 |

Blocking assay by IL-2 receptor α- and/or β-chain Ab was performed. Simultaneous use of both Abs inhibited the proliferation of clones stimulated with IL-2 and IL-4. Data of the representative TIL clone are shown.

IL-2 receptor from 12 500 to 15 500 molecules per cell. Similarly, IL-4 increased IL-2 binding to the low-affinity IL-2 receptor from 49 000 to 67 000 IL-2 molecules per cell. However, IL-4 did not change the slope of the high- or low-affinity IL-2 receptor binding curves.

### *Internalisation of IL-2*

To analyse the binding of IL-2 to the TIL clones further, internalisation of IL-2 was studied. Data of representative TIL clones are shown in Table III. At early time points (10 and 30 min) following incubation with IL-4, the internalisation of IL-2 was enhanced as compared with incubation with IL-2 alone. However, at later time points (60 and 120 min) of incubation, there was no difference between IL-2 alone and IL-2 plus IL-4 in the amount of IL-2 internalised. Moreover, after incubation with IL-4 for only 10 min, over 75% of the IL-2 was internalised. This demonstrates that IL-4 augments the internalisation of IL-2 at an early phase.

**Figure 2** Binding assay and Scatchard analysis of TIL clone stimulated with IL-4. IL-4 up-regulated the low- and high-affinity IL-2 receptors without altering the slope of either receptor. Results were obtained using the representative TIL clone, 2D10.

### Discussion

It has been reported that IL-4 augments the specific cytotoxic activity against autologous melanoma cells and other types of tumour cells (Kawakami *et al.*, 1988, 1993). We have already demonstrated that IL-4 accelerates the proliferation of IL-2-activated TILs without altering the cytotoxic activity against autologous adenocarcinoma cells, and IL-4 does not alter the phenotypes of TILs (Tsunoda *et al.*, 1992). However, the mechanism of IL-4 has remained unclear. In order to clarify the mechanism of IL-4, we first established clones of TILs. $CD4^+$, $CD8^+$ and $CD4^+CD8^+$ (double positive) T-cell clones were established from freshly isolated TILs. About 3% of T cells in peripheral blood are double-positive T cells (Blue *et al.*, 1985). Double-positive T cells are thought to be an intermediate phenotype between immature double-negative T cells and mature single-positive (CD4 or CD8) T cells during the differentiation of T cells in the thymus (McPhee *et al.*, 1979; Penit, 1986). Our studies (Yamaue *et al.*, 1990) and those of others (Whiteside *et al.*, 1986; Ebert *et al.*, 1989; Shimizu *et al.*, 1990; Whitford *et al.*, 1990; Viale *et al.*, 1990) have not observed a subpopulation of double-positive T cells in TILs. In our system, IL-4 was used to establish TIL clones and it was thought that IL-4 augments the expression of the CD8 molecule in $CD4^+$ T cells (Paliard *et al.*, 1988). This might be the reason why we could establish double-positive T-cell clones.

The cytokine production of these TIL clones was measured. IL-1, IFN-γ and TNFα were produced from these TIL clones (data not shown). It may be indicated that IL-4 augments the cytokine network system (Lorre *et al.*, 1990).

Next, by using three different T-cell clones, proliferation

**Table III** Internalisation of IL-2 molecule in TIL clones incubated with IL-4

| TIL clone | In vitro treatment | 0 | 10 | 30 | 60 | 120 |
|---|---|---|---|---|---|---|
| | | *Time course (min) of [$^{125}$I]IL-2 ($\times 10^3$c.p.m.)* | | | | |
| IF7 | IL-2 | 2.7 | 4.1 | 4.5 | 7.2 | 8.1 |
| | IL-2+IL-4 | 2.8 | 6.9 | 7.1 | 7.7 | 9.3 |
| 2F4 | IL-2 | 2.5 | 3.4 | 3.3 | 5.2 | 6.1 |
| | IL-2+IL-4 | 2.5 | 6.4 | 6.8 | 5.6 | 7.2 |
| 2D10 | IL-2 | 4.2 | 5.0 | 6.0 | 9.5 | 11.2 |
| | IL-2+IL-4 | 4.2 | 8.1 | 9.3 | 9.9 | 12.0 |

IL-4 augmented the internalisation of IL-2 during the early phase (10 and 30 min). Over 75% of internalisation was acheived by 30 min. Representative data were shown. The value of time 0 means the background proliferation.

induced by IL-4 and/or IL-2 was measured. IL-4 alone augmented proliferation of TIL clones irrespective of phenotype, and furthermore, IL-4 combined with IL-2 enhanced the proliferation of every TIL clone in comparison with IL-2 alone. These results indicate two important points. First, IL-4 augments the proliferation of TILs at the clonal level independent of their phenotype. Second, the augmentation of IL-4 combined with IL-2 is synergistic. In the present report, we focused on the mechanism by which IL-4 enhanced the proliferation induced with IL-2. In this mechanism, IL-2 and IL-2 receptor system were thought to be crucial (Fernandez-Botran *et al.*, 1989; Kawakami *et al.*, 1989; Ishikawa *et al.*, 1991). In our studies, a blocking assay using anti-IL-2 receptor α-chain antibody (CD25) and β-chain antibody (TU27) was performed on representative clones. IL-2 receptor α chain alone (low affinity) and β chain alone (intermediate) did not inhibit proliferation in comparison with control antibody. However, simultaneous addition of both IL-2 receptor antibodies inhibited proliferation. Interestingly, the inhibition was similar in clones cultured with IL-2 alone or with IL-2 plus IL-4. It is suggested that the augmentation of IL-4 is related to high-affinity IL-2 receptor. However, cell surface expression of IL-2 receptor α-chain, β-chain and IL-4 receptor by these clones was analysed by flow cytometry. There was no difference in receptor expression between cells stimulated with IL-2 alone or IL-2 plus IL-4 (data not shown).

Furthermore, the IL-2 binding assay and Scatchard analysis at clonal level showed that IL-4 did not change the value of $K_d$ of the high-affinity IL-2 receptor (200 pM) and the low-affinity IL-2 receptor (2.8 nM) in comparison with IL-2 alone. These data suggest that IL-4 does not change the functional characteristics of the IL-2 receptor. On the other hand, IL-4 increased the binding amount of IL-2 molecule on the cell surface at both the IL-2 receptors. It is especially important that IL-4 increases the binding of IL-2 on the high-affinity IL-2 receptor, since this receptor is thought to be related to signal transduction (Yagita *et al.*, 1989). This may be the mechanism for the synergistic effect of IL-4 on proliferation.

The increase of binding IL-2 molecule on TILs exposed to IL-4 prompted us to examine the internalisation of IL-2, which depends on the latter receptor (Fujii *et al.*, 1986; Siegel *et al.*, 1987; Robb *et al.*, 1987; Yoshimoto *et al.*, 1990). It was found that internalisation of IL-2 was augmented by IL-4 at the early incubation time points (10 and 30 min). Thus, the enhanced internalisation of IL-2 induced by IL-4 may accelerate delivery signals of transduction that is followed by the heightened proliferation of TILs. The combined effects that IL-4 has on up-regulation of the high-affinity IL-2 receptor, increasing the amount of IL-2 bound per TIL, and enhancement of internalisation of IL-2 together lead to augmentation of TIL proliferation.

Recently, IL-2 receptor γ chain has been sequenced and analysed in detail (Takeshita *et al.*, 1992). High-affinity IL-2 receptor was clarified to consist of α-, β- and γ-chain, and the γ-chain was shown to be crucial to signal transduction (Nakamura *et al.*, 1993). Moreover, the IL-2 receptor γ-chain has high homology to the IL-4 receptor (Kondo *et al.*, 1993). IL-2 receptor γ-chain may play an important role in the augmentation of TIL proliferation caused by IL-4. We have shown at the clonal level that IL-4 augmented the proliferation of TIL clones via the IL-2 receptor. At present, we are now attempting to analyse the involvement of the IL-2 receptor γ-chain in the stimulation of proliferation produced by IL-4.

The expression of IL-4 receptor was analysed by radioimmunoassay. There was no significant difference by stimulation with IL-4 (data not shown). However, it is possible that the phenomena reported reflect some inside-out modulation of surface IL-2 receptor mediated via the IL-4/IL-4 receptor system.

**Acknowledgements**
This work was supported in part by grants-in-aid from the Japanese Foundation for multidisciplinary treatment of cancer. We wish to thank Dr F James Primus, Beckman Research Institute of the City of Hope, for his valuable criticism and review of the manuscript.

### References

BLUE M, DALEY FJ, LEVINE H AND SCHLOSSMAN FS. (1985). Coexpression of T4 and T8 on peripheral blood T cells demonstrated by two-color fluorescence flow cytometry. *J. Immunol.*, **134**, 2281-2286.

EBERT CE, BROLIN ER AND ROBERTS IA. (1989). Characterization of activated lymphocytes in colon cancer. *Clin. Immunol. Immunopathol.*, **50**, 72-81.

FERNANDEZ-BOTRAN R, SANDERS VM AND VITETTA ES. (1989). Interactions between receptors for interleukin 2 and interleukin 4 on lines of helper T cells (HT-2) and B lymphoma cells (BCL1). *J. Exp. Med.*, **169**, 379-391.

FUJII M, SUGAMURA K, SANO K, NAKAI M, SUGITA K AND HIMURA Y. (1986). High-affinity receptor-mediated internalization and degradation of interleukin 2 in human T cells. *J. Exp. Med.*, **163**, 550-562.

HOROHOV DW, CRIM JA, SMITH PL AND SIEGEL JP. (1988). IL-4 (B cell-stimulatory factor 1) regulates multiple aspects of influenza virus-specific cell-mediated immunity. *J. Immunol.*, **141**, 4117-4223.

HOWARD M, FARRAR J, HILFIKER M, JOHNSON B, TAKATSU K, HAMAOKA T AND PAUL WE. (1982). Identification of a T cell-derived B cell growth factor distinct from interleukin 2. *J. Exp. Med.*, **155,** 914–923.

ISHIKAWA T, UCHIYAMA T, KAMIO M, ONISHI R, KODAKA T AND OKUMA M. (1991). IL-4 down-regulates IL-2 receptor p75 by accelerating its endocytosis. *Int. Immunol.*, **3,** 517–525.

IWAHASHI M, TANIMURA H, YAMAUE H, TSUNODA T, TANI M, NOGUCHI K, MIZOBATA S, HOTTA T AND TAMAI M. (1993). An autoreactive T cell clone generated in the autologous mixed lymphocytes reaction in a patient with gastric carcinoma. *J. Clin. Lab. Immunol.*, **40,** 29 37.

KAWAKAMI Y, ROSENBERG SA AND LOTZE MT. (1988). Interleukin 4 promotes the growth of tumor-infiltrating lymphocytes cytotoxic for human autologous melanoma. *J. Exp. Med.*, **168,** 2183–2191.

KAWAKAMI Y, CUSTER MC, ROSENBERG SA AND LOTZE TM. (1989). IL-4 regulates IL-2 induction of lymphokine-activated killer activity from human lymphocytes. *J. Immunol.*, **142,** 3452–3461.

KAWAKAMI Y, HAAS GP AND LOTZE MT. (1993). Expansion of tumor-infiltrating lymphocytes from human tumors using the T-cell growth factors interleukin-2 and interleukin-4. *J. Immunother.*, **14,** 336–347.

KONDO M, TAKESHITA T, ISHII N, NAKAMURA M, WATANABE S, ARAI K AND SUGAMURA K. (1993). Sharing of the interleukin-2 (IL-2) receptor gamma chain between receptors for IL-2 and IL-4. *Science*, **262,** 1874–1877.

LORRE K, DAMME VJ AND CEUPPENS LJ. (1990). A bidirectional regulatory network involving IL 2 and IL 4 in the alternative CD2 pathway of T cell activation. *Eur. J. Immunol.*, **20,** 1569–1575.

MCPHEE D, PYE J AND SHORTMAN K. (1979). The differentiation of T lymphocytes. Evidence for intrathymic death of most thymocytes. *Thymus*, **1,** 151–162.

MITCHELL LC, DAVIS LS AND LIPSKY PE. (1989). Promotion of human T lymphocyte proliferation by IL-4. *J. Immunol.*, **142,** 1548–1557.

MOSMANN TR, CHERWINSKI H, BOND MW, GIEDLIN MA AND COFFMAN RL. (1986). Two types of murine helper T cell clone: I. Definition according to profiles of lymphokine activities and secreted proteins. *J. Immunol.*, **136,** 2348–2357.

MULE JJ, SMITH CA AND ROSENBERG SA. (1987). Interleukin 4 (B cell stimulatory factor 1) can mediate the induction of lymphokine-activated killer cell activity directed against fresh tumor cells. *J. Exp. Med.*, **166,** 792–797.

NAGLER A, LANIER LL AND PHILLIPS JH. (1988). The effect of IL-4 on human natural killer cells. A potent regulator of IL-2-activation and proliferation. *J. Immunol.*, **142,** 2349–2351.

NAKAMURA M, ASAO H, TAKESHITA T AND SUGAMURA K. (1993). Interleukin-2 receptor heterotrimer complex and intracellular signaling. *Semin. Immunol.*, **5,** 309–317.

PALIARD X, MALEFIJT RW, DE VRIES JE AND SPITZ H. (1988). Interleukin-4 mediates CD8 induction on human $CD4^+$ T-cell clones. *Nature*, **335,** 642–644.

PAUL WE AND OHARA J. (1987). B-cell stimulatory factor-1/interleukin-4. *Annu. Rev. Immunol.*, **5,** 429–459.

PENIT C. (1986). *In vitro* thymocyte maturation. BUdR labeling of cycling thymocytes and phenotypic analysis of their progeny support the single lineage model. *J. Immunol.*, **137,** 2115–2121.

ROBB JR AND GREEN CW. (1987). Internalization of interleukin 2 is mediated by the $\beta$ chain of the high-affinity interleukin 2 receptor. *J. Exp. Med.*, **165,** 1201–1206.

ROBB JR, GREEN CW AND RUSK MC. (1984). Low and high affinity cellular receptors for interleukin 2. *J. Exp. Med.*, **160,** 1126–1146.

ROSENBERG SA, SEPIESS P AND LATRENIERE R. (1986). A new approach to the adoptive immunotherapy of cancer with tumor-infiltrating lymphocytes. *Science*, **233,** 1318–1321.

SHIMIZU Y, IWATSUKI S, HERBERMAN BR AND WHITESIDE LT. (1990). Clonal analysis of tumor-infiltrating lymphocytes from human primary and metastatic liver tumors. *Int. J. Cancer*, **46,** 878–883.

SIEGEL JP, MICHAEL S, SMITH PL AND LEONARD WJ. (1987). The IL-2 receptor $\beta$ chain (p70): role in mediating signals for LAK, NK, and proliferative activities. *Science*, **238,** 75–78.

SPITS H, YSSEL H AND TAKEBE T. (1987). Recombinant interleukin-4 promotes the growth of human T cells. *J. Immunol.*, **139,** 1142–1147.

SWAIN SL, MCKENZIE DT, DUTTON RW, TONKONOGY SL AND ENGLISH M. (1988). The role of IL-4 and IL-5: characterization of a distinct helper T cell subset that makes IL-4 and IL-5 (Th2) and requires priming before induction lymphokine secretion. *Immunol. Rev.*, **102,** 77–105.

TAKESHITA T, ASAO H, OHTANI K, ISHII N, KUMAKI S, TANAKA N, NAKAMURA M AND SUGAMURA K. (1992). Cloning of the gamma chain of the human IL-2 receptor. *Science*, **257,** 379–382.

TOPALIAN SL, SOLOMON D, AVIS FP, CHANG AE, FREEEKSEN DL, MARSTON LINEHAN W, LOTZE MT, ROBERTSON CN, SEIPP CA, SIMON P, SIMPSON CG AND ROSENBERG SA. (1988). Immunotherapy of patients with advanced cancer using tumor-infiltrating lymphocytes and recombinant interleukin 2: a pilot study. *J. Clin. Oncol.*, **6,** 839–853.

TSUNODA T, TANIMURA H, YAMAUE H, IWAHASHI M, TANI M, TAMAI M, ARII K AND NOGUCHI K. (1992*a*). The promotive effect of interleukin 4 with interleukin 2 in the proliferation of tumor-infiltrating lymphocytes from patients with malignant tumor. *Biotherapy*, **4,** 9–15.

TSUNODA T, TANIMURA H, YAMAUE H, IWAHASHI M, TANI M, TAMAI M, ARII K AND NOGUCHI K. (1992*b*). *In vitro* augmentation of the cytotoxic activity of peripheral blood mononuclear cells and tumor-infiltrating lymphocytes by famotidine in cancer patients. *Int. J. Immunopharmacol.*, **14,** 75–81.

VIALE M, FERRIN S, SERRANO S, ARDIZZONI A AND NICOLIN A. (1990). Peripheral blood and tumor-infiltrating lymphocytes in non-small cell lung cancer: analysis at the population and clonal level. *Tumori*, **76,** 484–488.

WHITESIDE LT, MIESCHER S, MORETTA L AND FLIEDNER V. (1986). Clonal analysis and in situ characterization of human breast carcinomas. *Cancer Immunol. Immunother.*, **23,** 169–178.

WHITFORD P, MALLON AE, GEORGE DW AND CAMPBELL M. (1990). Flow cytometric analysis of tumour-infiltrating lymphocytes in breast cancer. *Br. J. Cancer*, **62,** 971–975.

YAGITA H, NAKATA M, AZUMA A, NITTA T, TAKESHITA T, SUGAMURA K AND OKUMURA K. (1989). Activation of peripheral blood T cells via the p75 interleukin 2 receptor. *J. Exp. Med.*, **170,** 1445–1450.

YAMAUE H, TANIMURA H, TSUNODA T, IWAHASHI M, TANI M, TAMAI M. AND INOUE M. (1990). Functional and phenotypic analyses of interleukin 2-activated tumor-infiltrating lymphocytes. *Biotherapy*, **2,** 247–259.

YOSHIMOTO T, NAKANISHI K, MATSUI K, HIROSE S, HIROISHI K, TANAKA T, HADA T, HAMAOKA T AND HIGASHIO K. (1990). IL-5 up-regulates but IL-4 down-regulates IL-2 R expression on a cloned B lymphoma line. *J. Immunol.*, **144,** 183–190.